# Frontotemporal cortical activity in Chinese male alcohol-dependent patients during a verbal fluency task: a functional near-infrared spectroscopy study

**DOI:** 10.3389/fpsyt.2026.1827350

**Published:** 2026-06-16

**Authors:** Suilin Jia, Jie Liu, Jiaqi Zheng, Meizhi Zheng, Lijun Wang, Wei Jin, Chengqian Jin, Yuan Li, Hong Chen, Ke Zhao

**Affiliations:** 1 Department of Behavioral Medicine, Wenzhou Seventh People’s Hospital, Wenzhou, China; 2 School of Mental Health, Wenzhou Medical University, Wenzhou, China; 3 Department of Psychiatry, First Affiliated Hospital of Wenzhou Medical University, Wenzhou, China; 4 The Affiliated Kangning Hospital of Wenzhou Medical University, Zhejiang Provincial Clinical Research Center for Mental Health, Wenzhou, China

**Keywords:** alcohol-dependent, near-infrared spectroscopy, prefrontal cortex, rDLPFC, verbal fluency task

## Abstract

**Introduction:**

Alcohol dependence is associated with alterations in prefrontal cortical function; however, previous functional near-infrared spectroscopy studies using verbal fluency tasks have produced inconsistent findings. This study examined differences in task-related cortical activation between Chinese male patients with alcohol dependence and healthy controls.

**Methods:**

Thirty-one right-handed male inpatients with alcohol dependence and 31 healthy male controls completed a Mandarin verbal fluency task. A 52-channel functional near-infrared spectroscopy system was used to measure task-related changes in oxygenated hemoglobin concentrations. Channel-wise Δβ values were compared between the groups using independentsamples t-tests with correction for multiple comparisons.

**Results:**

The groups did not differ significantly in age or years of education. Compared with healthy controls, patients with alcohol dependence exhibited greater oxygenated hemoglobin activation in channel 23, which primarily covered the right dorsolateral prefrontal cortex and partially overlapped with the right inferior frontal gyrus (t = 3.315, FDR-adjusted q = 0.104). No significant between-group differences were observed in the other channels.

**Discussion:**

Increased activation in the right dorsolateral prefrontal cortex and right inferior frontal gyrus may reflect compensatory recruitment of neural resources or increased engagement of cognitive-control and emotion-regulation systems. Functional near-infrared spectroscopy may be a useful tool for assessing cortical function in alcohol dependence, although larger, mixed-sex, and cross-linguistic studies are required to validate these findings.

## 1 Introduction

Alcohol dependence (AD), also known as alcohol use disorder (AUD), is a widespread mental health issue with global prevalence. Alcohol consumption is associated with substantial global health burden and premature mortality (1). In China, the prevalence and lifetime prevalence of alcohol dependence are 2.2% and 3.7%, respectively, similar to figures reported in Western countries (2). However, drinking patterns in China differ notably from those in Western countries, particularly the frequent alcohol consumption by middle-aged men as a means of socialization. As a result, alcohol-related problems in China are more common among married, working men in their 30s and less common among women (3) The prevalence of alcohol dependence has notably increased, particularly following the COVID-19 outbreak (4). Alcohol dependence thus presents an urgent public health challenge.

Alcohol dependence not only causes physical harm but also leads to alterations in brain function and structure, negatively affecting cognitive performance (5). Research indicates that damage to the prefrontal cortex is a key factor in cognitive dysfunction and behavioral abnormalities in individuals with alcohol dependence (6). Furthermore, the extent of prefrontal cortex damage may be associated with the risk of relapse in these individuals. Specifically, alcohol-dependent patients who relapse after treatment exhibit significant reductions in the total volume of the prefrontal cortex (7, 8) and its subregions (9) compared to those who maintain abstinence. Beyond cognitive dysfunction, alcohol dependence is closely associated with emotional dysregulation and alexithymia, both of which may contribute to maladaptive drinking behaviors and relapse vulnerability. Emotional dysregulation has been linked to substance misuse and addictive behaviors (10), while alexithymia, characterized by difficulty identifying and describing emotional states, has been shown to predict relapse outcomes in patients with alcohol use disorder (11). These affective disturbances are particularly relevant to the present study because the dorsolateral prefrontal cortex is involved not only in executive control but also in the cognitive regulation of emotion (12). Recent neuroimaging studies continue to reveal changes in brain function in alcohol-dependent patients. Functional Magnetic Resonance Imaging (fMRI) techniques quantify the coherence and strength of functional connectivity (FC) between different brain regions (13–15). One study (16) found that FC was generally lower in alcohol-dependent individuals compared to controls, whereas another study (17) observed higher FC across multiple networks. In cognitive tasks, diminished functional connectivity between the right prefrontal and insula regions was found to be associated with reduced memory function in alcohol-dependent patients (18). In contrast, Jansen et al. (19) noted that the integrity of prefrontal white matter connectivity was positively correlated with cognitive flexibility.

In recent years, although fMRI offers superior spatial resolution compared to functional Near-Infrared Spectroscopy (fNIRS), fNIRS excels in temporal resolution, providing more accurate and comprehensive information on functional signals in brain regions. As an emerging functional brain imaging technique, fNIRS is recognized for its low cost, portability, lack of noise interference, non-invasiveness, and insensitivity to subject movement, making it a favored tool among researchers (20). During cognitive activities, the brain increases blood oxygen levels through neurovascular coupling: neuronal activity leads to a rapid increase in oxygenated hemoglobin (Oxy-Hb) concentration and a corresponding decrease in deoxygenated hemoglobin (Deoxy-Hb) concentration in the active brain regions. Based on this principle, fNIRS reflects neural activity (21). Currently, fNIRS is widely used in clinical research on various mental disorders, such as schizophrenia, bipolar disorder, and depression (22). However, its application in alcohol dependence research remains relatively limited.

Most fNIRS studies on alcohol dependence have measured brain function during the Verbal Fluency Test (VFT). The VFT is commonly paired with fNIRS due to its ease of administration, short duration, and minimal equipment or space requirements. The VFT has been used in conjunction with near-infrared spectroscopy (NIRS) to assess prefrontal cortex (PFC) activation in both healthy individuals and those with mental illnesses (23). Schecklmann et al. (24) found that, compared to healthy participants, alcohol-dependent patients exhibited reduced activation, with activation more restricted to subfrontal regions. Dresler et al. (25) conducted a cross-sectional study and reported that three patient groups—acute withdrawal, detoxification, and moderate—showed reduced frontal activation during fluency tasks compared to healthy controls. A significant linear trend was observed, with activation in the withdrawal group being closer to normal and not significantly different from that of controls. Brain activation increased over time in the detoxification group. However, in a subsequent study, no significant differences were found in behavioral performance or Oxy-Hb changes during the VFT between the detoxification and relapse groups (26).

Given the limited number of fNIRS studies on alcohol dependence and the inconsistent findings, the present study aims to examine differences in the hemodynamic response in the prefrontal cortex (PFC) during the Verbal Fluency Task (VFT) in alcohol-dependent patients. This study seeks to better understand the differences in neural activity between alcohol-dependent patients and healthy controls, and to identify potential neurobiological markers associated with alcohol dependence. It is hypothesized that alcohol-dependent patients will exhibit reduced prefrontal activation during the VFT compared to healthy controls.

## 2 Methods

### 2.1 Participants

A total of 31 right-handed male inpatients from the Seventh People’s Hospital of Wenzhou and 31 healthy male controls were included in the study. The healthy control group consumed no more than 10g of alcohol per week. All patients met the diagnostic criteria for alcohol dependence as outlined in the 5th edition of the Diagnostic and Statistical Manual of Mental Disorders (DSM-V). fNIRS assessments were conducted after withdrawal symptoms had resolved.

The study was approved by the Ethics Committee of the Seventh People’s Hospital of Wenzhou. Written informed consent was obtained from all participants following a thorough explanation of the procedures.

### 2.2 VFT

**Figure 1:**
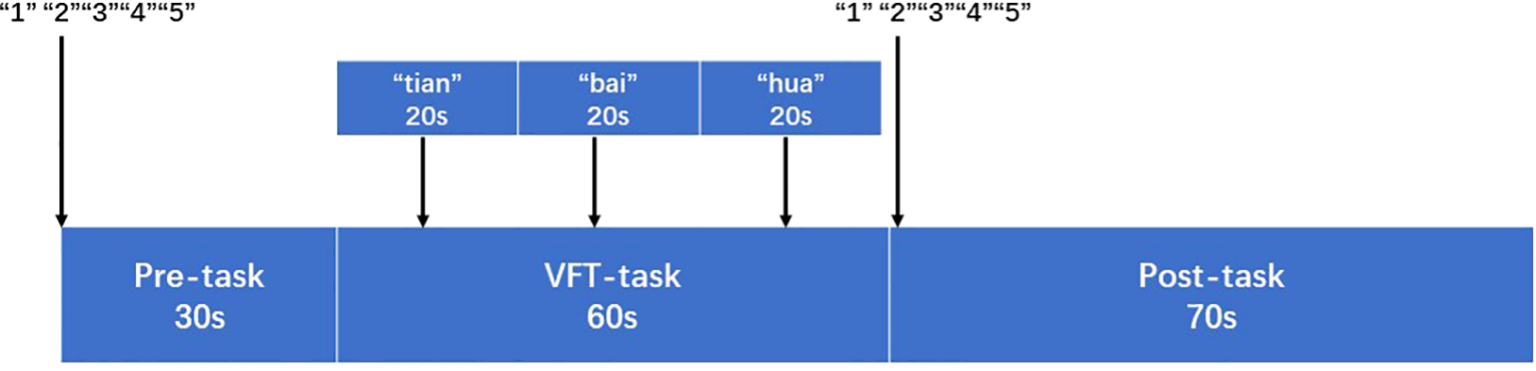
The verbal fluency trials consist of a 30-second baseline period, followed by a 60-second task phase and a 70-second rest period after the task.

The VFT was adapted from Wei et al. (27). Throughout the assessment, participants were seated in a quiet environment, instructed to keep their eyes open, avoid excessive movement, and focus on a cross displayed on the screen. The procedure consisted of a 30-second preparation phase, a 60-second active task phase, and a 70-second recovery phase. During the preparation and recovery phases, participants recited “1, 2, 3, 4, 5” in a continuous cycle. In the task phase, they were asked to generate as many phrases as possible starting with a given character (e.g., “天,” “白,” “花,” corresponding to sky, white, and flower). The task included three different cue characters, each presented for 20 seconds during the 60-second active phase. The experimental procedure is depicted in **Figure 1**.

### 2.3 The fNIRS measurement

**Figure 2:**
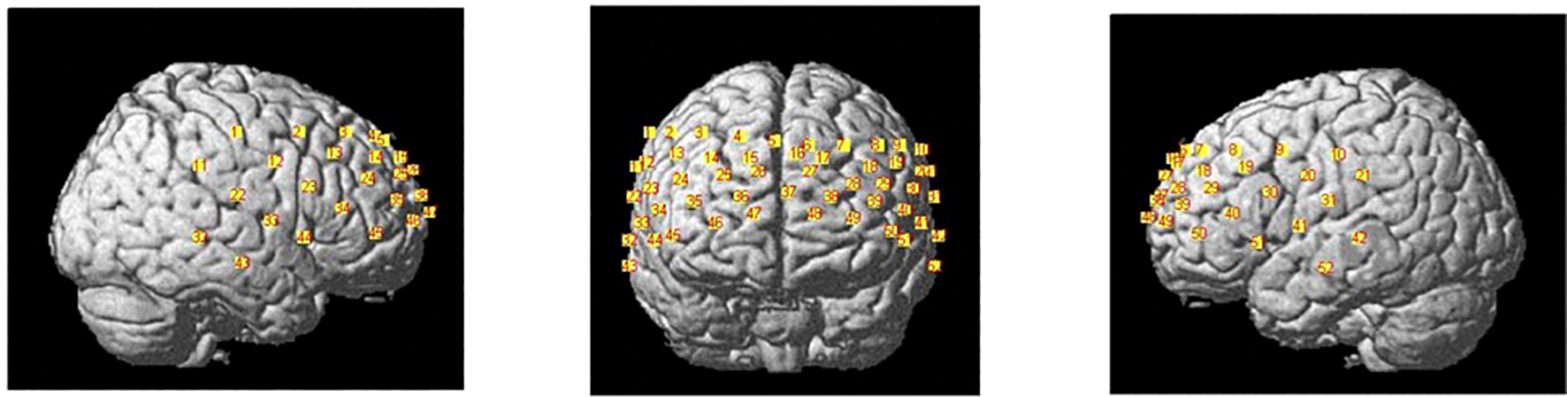
Channel layout of fNIRS cap.

The ETG-4000, manufactured by Hitachi Medical Co. in Tokyo, Japan, is a 52-channel fNIRS device that uses two wavelengths of near-infrared light (695 nm and 830 nm) to assess changes in blood flow in the prefrontal and supratemporal brain regions. It is equipped with 16 detectors and 17 emitters arranged in a 3 × 11 matrix, providing 52 channels, in accordance with the international 10–20 electrode placement system. This configuration ensures comprehensive coverage of the hemodynamic response across the bilateral prefrontal cortex as well as the anterior and superior regions of the temporal cortex. The channel layout of the fNIRS cap used in this study is shown in **Figure 2**. The NIR system records concentrations of oxygenated hemoglobin (HbO), deoxygenated hemoglobin (HbR), and total hemoglobin (HbT), which reflect neural activity through neurovascular coupling and task-evoked changes in cerebral blood flow. HbO is typically more sensitive to functional activation, closely following local increases in blood flow upon neuronal activation, while HbR provides complementary information regarding oxygen extraction. Signal characteristics can vary across brain regions depending on cortical depth, vascular density, and local hemodynamic response properties (28–30). Therefore, subsequent analyses in this study are based on the modified Beer-Lambert law for HbO (31).

### 2.4 The fNIRS signal analysis

The fNIRS data are analyzed using the NIRS-SPM toolbox (32), a statistical processing software developed based on MATLAB. This toolbox employs a General Linear Model (GLM), a standard regression technique widely used for both fMRI data evaluation and fNIRS data interpretation. The GLM framework includes the variables Y, X, β, and ϵ, expressed as Y = βX + ϵ. Where Y represents the actual fNIRS measurements, including both time series and probe channels. X corresponds to the expected values derived from the experimental protocol, which are obtained by integrating the hemodynamic response function (HRF) with the event timeline. The coefficient β indicates the extent to which the Y signal is influenced by the experimental design matrix X, reflecting the degree of cortical activity induced by the VFT in this study. ϵ accounts for residual variance. The raw fNIRS data are preprocessed using the HRF and discrete cosine transform (DCT) methods to reduce noise, including signal drift and motion artifacts (33). Based on previous research, oxyhemoglobin (HbO) levels are considered a more sensitive and consistent metric in fNIRS analysis compared to deoxyhemoglobin (HbR) concentrations (34).

### 2.5 Statistical analysis

The change in oxyhemoglobin concentration, represented by the △β value (the difference between the VFT β value and the baseline β value), serves as an indicator of cortical activation levels during the VFT (35). Data analysis is performed using SPSS version 22.0. Continuous variables, including age, years of education, and channel-wise △β values, were compared between the alcohol-dependent and healthy control groups using independent-samples t-tests. All statistical tests were conducted with a two-tailed approach. To control for multiple comparisons across the fNIRS channels, p-values were adjusted using the false discovery rate (FDR) method (36). Given the exploratory nature of this study and the limited sample size, the FDR threshold was set at q < 0.2 to balance false-positive control and sensitivity for detecting potential hemodynamic differences across multiple channels (37, 38).

## 3 Results

### 3.1 Demographic characteristics of all participants

The alcohol-dependent (AD) group consisted of 31 males with a mean age of 43.74 years (SD = 8.37) and an average of 11.16 years of education (SD = 2.49), while the healthy control (HC) group also included 31 males with a mean age of 48.10 years (SD = 9.54) and 11.00 years of education (SD = 2.68). There were no significant differences between the groups in age or years of education. See **Table 1** for full demographic and clinical characteristics.

**Table 1:** Demographic and clinical characteristics of alcohol-dependent (AD) and healthy control (HC) groups.

Variable	AD (n = 31)	HC (n = 31)	t	P
Age, year	43.74 ± 8.37	48.10 ± 9.54	-1.91	>0.05
Sex (male)	31	31	—	—
Years of education	11.16 ± 2.49	11.00 ± 2.68	0.25	>0.05
Duration of drinking, year	17.03 ± 8.77	—	—	—
Time of admissions, day	24.52 ± 16.05	—	—	—
First-episode cases, n (%)	16 (51.61)	—	—	—
Severity of alcohol dependence, n (%)				
Moderate	5 (16.13)	—	—	—
Severe	26 (83.87)	—	—	—
Medication use, n (%)				
Withdrawal-management medications/benzodiazepines	31 (100.0)	—	—	—
Antipsychotics	7 (22.58)	—	—	—
Mood stabilizers	5 (16.13)	—	—	—
Antidepressants	6 (19.35)	—	—	—
No additional psychotropic medication	3 (9.68)	—	—	—

Values expressed as mean ± standard deviation or number of participants.

### 3.2 Hemodynamic response during the VFT

**Figure 3:**
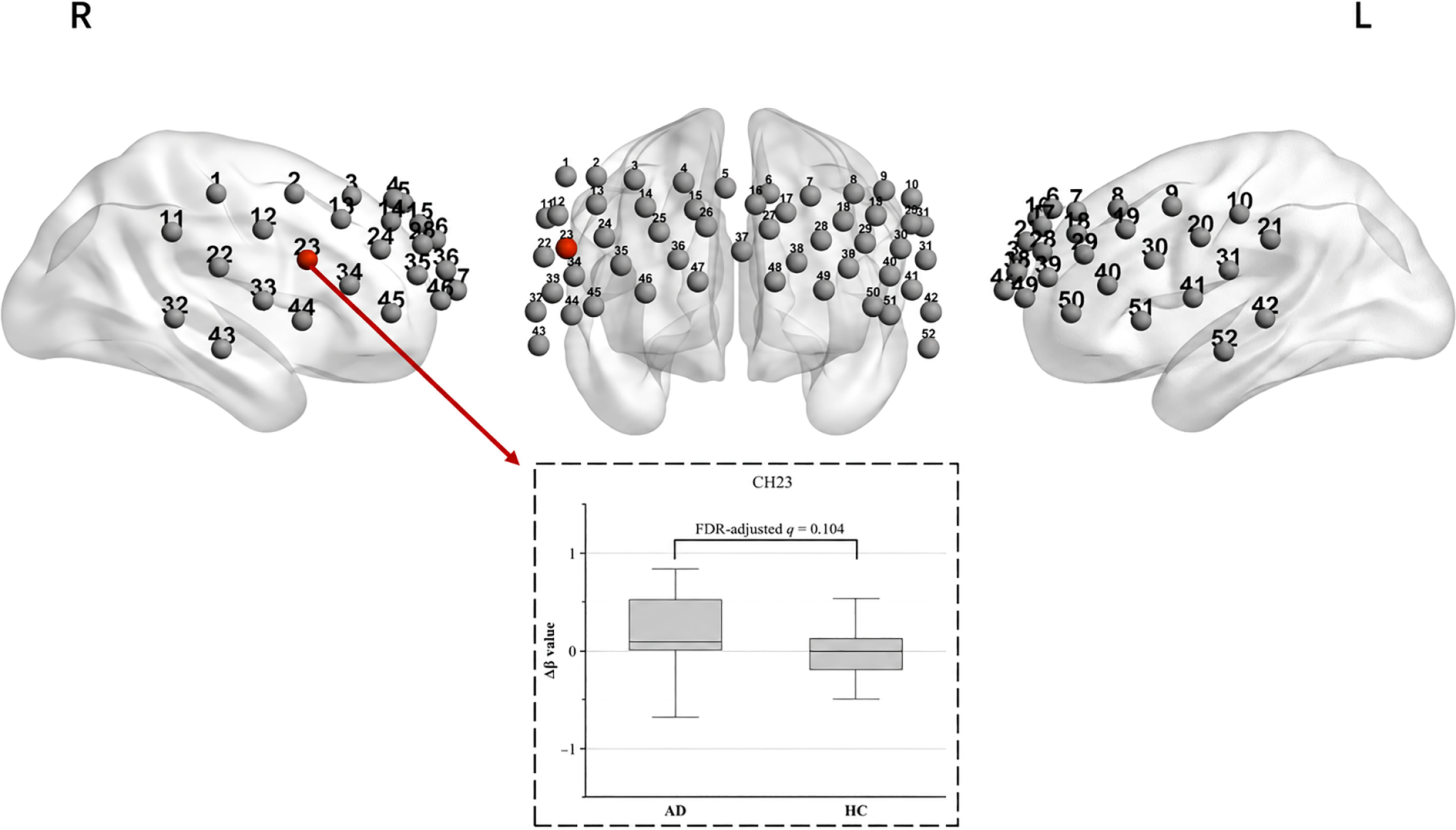
During the VFT, channel 23, shown in red, exhibited a higher mean Δβ value of oxygenated hemoglobin in the alcohol-dependent group than in the healthy-control group.

Independent samples t-test analysis revealed that the Δβ value for channel 23, located over the right lateral prefrontal cortex, primarily covering the dorsolateral prefrontal cortex (BA9/46) and partially overlapping with the right inferior frontal gyrus (BA44/45, pars opercularis and pars triangularis), was significantly higher in the alcohol-dependent group compared to the healthy control group (t = 3.315, FDR-adjusted *q* = 0.104, **Figure 3**), No significant differences were observed in the Δβ values of other channels between the two groups.

## 4 Discussion

In the present study, fNIRS was used to examine cortical activation during the VFT in Chinese male patients with alcohol dependence. The main finding was that patients with alcohol dependence showed increased activation in channel 23, which primarily covers the right dorsolateral prefrontal cortex (rDLPFC) with partial overlap with the right inferior frontal gyrus (rIFG), compared with healthy controls.

Previous findings on brain activity in patients with alcohol dependence during the VFT have been inconsistent. German studies reported reduced prefrontal cortex (PFC) activation in patients with alcohol dependence compared with healthy controls during the VFT (24, 25), whereas a Japanese study found no significant group difference in brain activation during the same task (26). These discrepancies may be related to several factors, including psychotropic medication use, illness duration, relapse history, sample characteristics, and differences in language background and VFT paradigms.

Language-related differences may be particularly relevant when interpreting VFT-related cortical activation. Previous multichannel fNIRS evidence has demonstrated language-specific cortical activation patterns during verbal fluency tasks; for example, Japanese VFT elicited activation patterns that differed from those typically reported in syllable- or alphabet-based language (39). A recent scoping review further highlighted that VFT paradigms in Chinese and Japanese clinical fNIRS settings may differ in task design, stimulus modality, and language typology, which may influence the interpretation of cortical activation patterns (40). In addition, unlike more established neuroimaging techniques such as fMRI and positron emission tomography, standardized protocols for fNIRS data acquisition and analysis remain limited. Therefore, differences in preprocessing strategies, channel selection, and statistical approaches across studies may also complicate direct comparisons and interpretation of findings (32, 41, 42).

The hyperactivation observed in channel 23 may be understood through multiple interacting mechanisms. First, as part of the cognitive control network, the rDLPFC and rIFG are involved in regulating negative emotions and suppressing inappropriate responses (12, 43). Functional imaging studies show that these regions are engaged during emotion regulation strategies such as reappraisal or suppression, exerting top-down control over limbic structures such as the amygdala (44, 45). chronic alcohol use may disrupt the balance between PFC regulatory circuits and limbic emotion-processing regions, thereby impairing the regulation of negative affect and craving (46). Therefore, the observed hyperactivation may reflect increased recruitment of PFC resources to regulate heightened negative affect and craving.

Second, chronic stress may further modulate PFC activation through stress-related neuroendocrine mechanisms. Stress and alcohol exposure can interact to dysregulate the HPA axis and weaken PFC-limbic regulatory networks, contributing to relapse vulnerability and impaired emotional control (46, 47). The hyperactivation in the rDLPFC and rIFG observed in our study is also broadly consistent with fNIRS evidence showing that psychosocial stress can elicit increased cortical activity in regions of the cognitive control network, including the bilateral dorsolateral prefrontal cortex (DLPFC) and inferior frontal gyrus (IFG), during the Trier Social Stress Test (48). Thus, increased activation in channel 23 may partly reflect stress-related engagement of PFC regulatory systems, although this interpretation remains speculative.

Third, compensatory recruitment may also play a role. Alcohol-dependent patients often exhibit structural brain changes, including reductions in hippocampal, thalamic, pallidal, and ventromedial nuclei volumes (49–51). In this context, the Stereotyped Task Accommodation and Compensation (STAC) framework suggests that increased PFC activity in response to high cognitive load, particularly in the presence of neural alterations, may partially reflect compensatory recruitment of additional neural resources (52). Consistent with this framework, additional frontal activation has been proposed to compensate for structural changes and reduced neural processing efficiency in other brain regions (53). Enhanced PFC activation has also been reported in alcohol-dependent patients during other cognitive tasks (19), and increased rIFG activation during alcohol-specific inhibition has been associated with heightened craving and better drinking-related outcomes (54).

Overall, these interpretations should be considered preliminary. Although increased activation in channel 23 may reflect emotion regulation, stress-related PFC engagement, or possible compensatory recruitment, these explanations remain speculative. Given the spatial limitations of fNIRS and the absence of direct measures of craving, stress, emotion regulation, and structural brain alterations in the present study, future studies should combine fNIRS with complementary neuroimaging methods such as fMRI, as well as behavioral and physiological measures, to clarify the functional significance of this activation pattern.

fNIRS offers practical advantages for assessing cortical function and may serve as a useful tool for evaluating alcohol dependence. Compared with conventional neuroimaging techniques such as fMRI and EEG, fNIRS is relatively cost- and time-efficient: its setup is rapid, requires minimal preparation, and allows repeated measurements within a short time frame, making it suitable for longitudinal or bedside assessments (55, 56). However, the application of fNIRS in alcohol dependence research remains at an early stage. Larger studies are needed to examine the broader neural networks involved in alcohol dependence and to determine whether fNIRS-derived indices can serve as reliable biomarkers for assessment or treatment monitoring.

The present study has several limitations. First, the relatively small sample size may have limited the ability to detect subtle group differences and reduces the stability of the findings. Second, the sample included only right-handed Chinese male inpatients with alcohol dependence after withdrawal symptoms had resolved, which limits the generalizability of the results. Although alcohol use disorders and alcohol-related disease burden are more common among men than women globally and in China (1, 2), the exclusion of women prevents us from determining whether similar activation patterns would be observed in female patients. This limitation is important because previous neuroimaging studies have reported sex-related differences in alcohol-related brain alterations, including PFC volume and broader structural brain change (57, 58). Future studies including both male and female participants are needed to clarify whether the observed activation pattern is generalizable across sexes.

Moreover, all participants completed a Mandarin-based VFT. Therefore, although we interpret increased activation in channel 23 as potentially related to alcohol dependence, we cannot exclude the possibility that this result was partly influenced by Mandarin-specific lexical retrieval or cognitive control demands. In addition, standard cognitive screening instruments, such as the Mini-Mental State Examination (MMSE) or Montreal Cognitive Assessment (MoCA), were not administered; therefore, pre-existing cognitive impairment cannot be ruled out. Future cross-linguistic, cross-cultural, and mixed-sex studies with larger samples and more comprehensive clinical assessments are needed to validate these findings. Despite these limitations, our study provides preliminary evidence that fNIRS may help identify cortical activation patterns relevant to the assessment and treatment of alcohol dependence.

## 5 Conclusion

This study demonstrates hyperactivation in channel 23, covering the rDLPFC with partial overlap of the rIFG, in alcohol-dependent patients during the Verbal Fluency Task. These findings likely reflect a combination of compensatory recruitment of neural resources and adaptive engagement of the cognitive control network in response to cognitive demands and chronic stress. The results are consistent with theories suggesting heightened prefrontal activity as a compensatory mechanism for structural brain changes associated with alcohol dependence. fNIRS is shown to be a promising tool for assessing cortical function in this population, although larger, cross-linguistic studies are needed to validate these findings and establish reliable biomarkers. Despite limitations, including a small sample size and restricted demographic representation, these results contribute to understanding the neural mechanisms underlying alcohol dependence and may inform future diagnostic and therapeutic strategies.

## Data Availability

The raw data supporting the conclusions of this article will be made available by the authors, without undue reservation.
